# Correlation between adherence rates measured by MEMS and self-reported questionnaires: a meta-analysis

**DOI:** 10.1186/1477-7525-8-99

**Published:** 2010-09-13

**Authors:** Lizheng Shi, Jinan Liu, Vivian Fonseca, Philip Walker, Anupama Kalsekar, Manjiri Pawaskar

**Affiliations:** 1 Department of Health Systems Management, School of Public Health and Tropical Medicine, Tulane University, New Orleans, Louisiana, USA; 2 Department of Medicine, School of Medicine, Tulane University, New Orleans, Louisiana, USA; 3 Rudolph Matas Library of the Health Sciences, Tulane University, New Orleans, Louisiana, USA; 4 Health Outcomes Research, Eli Lilly and Company, Indianapolis, Indiana, USA

## Abstract

**Purpose:**

It is vital to understand the associations between the medication event monitoring systems (MEMS) and self-reported questionnaires (SRQs) because both are often used to measure medication adherence and can produce different results. In addition, the economic implication of using alternative measures is important as the cost of electronic monitoring devices is not covered by insurance, while self-reports are the most practical and cost-effective method in the clinical settings. This meta-analysis examined the correlations of two measurements of medication adherence: MEMS and SRQs.

**Methods:**

The literature search (1980-2009) used PubMed, OVID MEDLINE, PsycINFO (EBSCO), CINAHL (EBSCO), OVID HealthStar, EMBASE (Elsevier), and Cochrane Databases. Studies were included if the correlation coefficients [Pearson (r_p_) or Spearman (r_s_)] between adherences measured by both MEMS and SRQs were available or could be calculated from other statistics in the articles. Data were independently abstracted in duplicate with standardized protocol and abstraction form including 1) first author's name; 2) year of publication; 3) disease status of participants; 4) sample size; 5) mean age (year); 6) duration of trials (month); 7) SRQ names if available; 8) adherence (%) measured by MEMS; 9) adherence (%) measured by SRQ; 10) correlation coefficient and relative information, including p-value, 95% confidence interval (CI). A meta-analysis was conducted to pool the correlation coefficients using random-effect model.

**Results:**

Eleven studies (N = 1,684 patients) met the inclusion criteria. The mean of adherence measured by MEMS was 74.9% (range 53.4%-92.9%), versus 84.0% by SRQ (range 68.35%-95%). The correlation between adherence measured by MEMS and SRQs ranged from 0.24 to 0.87. The pooled correlation coefficient for 11 studies was 0.45 (p = 0.001, 95% confidence interval [95% CI]: 0.34-0.56). The subgroup meta-analysis on the seven studies reporting r_p _and four studies reporting r_s _reported the pooled correlation coefficient: 0.46 (p = 0.011, 95% CI: 0.33-0.59) and 0.43 (p = 0.0038, 95% CI: 0.23-0.64), respectively. No differences were found for other subgroup analyses.

**Conclusion:**

Medication adherence measured by MEMS and SRQs tends to be at least moderately correlated, suggesting that SRQs give a good estimate of medication adherence.

## Background

Medical adherence is defined as the extent to which a patient's medication taking coincides with medical or health advice [1]. Despite the proven efficacy of prescription drugs in reducing illness symptoms and preventing or minimizing associated complications, adherence rates to long-term pharmacotherapy tend to be approximately 50%, regardless of the illness, regimen or measurement criteria [2,3]. In addition, the adherence rate varies with disease conditions, ranging from 15% to 93% as reported in the literature [4]. Failure to adhere to medication regimens in the United States may cost as much as $300 billion annually, mediated by ineffectiveness of treatment and worsening of disease progression to poor outcomes, disease complications, medication adverse events, hospitalizations and re-hospitalizations, emergency department visits, and even death [5].

Measuring patient adherence to prescribed therapies is a first step towards developing a greater understanding of the potential for non-adherence and adverse outcomes. Two methods often used for this purpose are medication event monitoring systems (MEMS) and self-reported questionnaires (SRQs) [6]. In spite of the availability of these measures, they present several technical challenges in measuring adherence. The MEMS is a medication vial cap that electronically records the date and time of bottle opening. It is also known as the "imperfect gold standard," [7] due to its recording effectiveness in measurement of patient adherence. However, it could be time consuming, expensive, resource intensive and may not be suitable for all medications/formulations. Alternatively, self-reported questionnaires (SRQs) could be a very convenient choice for certain study designs. However, SRQs are subject to measurement bias such as social desirability, recall bias, and response bias; there have been mixed reports about the accuracy of self-reported adherence [8,9]. Therefore, the accuracy in measuring medication adherence is uncertain for SRQs. This uncertainty further limits the credibility and validity of results obtained using SRQs. The previous literature reviews have focused on some qualitative work examining the correlation between SRQs and other measures such as pharmacy refill records, and interview [8-10]. Hence, it is vital to understand their associations relative to electronic measures of adherence such as MEMS. In addition, the economic implication of using alternative measures such as SRQs is also important as the cost of electronic monitoring devices is not covered by insurance, and thus these devices are not in routine use while self-reports are the most useful method in the clinical setting for practical interventions on non-adherence.

To advance the knowledge on relationships between different measurements, this study was the first study attempting to assess and quantify the correlation between MEMS and SRQs used for the measurement of medication adherence. Hence the objective of this study was to perform a meta-analysis to examine the correlation between MEMS and SRQs.

## Methods

### Study Selection

The literature search for monitoring devices citations from 1980-April 2009 was performed using search terms: patient compliance, medication adherence, treatment compliance, drug monitoring, drug therapy, electronic, digital, computer, monitor, monitoring, drug, drugs, pharmaceutical preparations, compliance, and medications. The search time frame was determined appropriately because the MEMS technology is available in 1980 s. We searched the following databases: PubMed, OVID MEDLINE, PsycINFO (EBSCO), CINAHL (EBSCO), OVID HealthStar, EMBASE (Elsevier), and Cochrane Databases of Systematic Reviews. The search was restricted to only human studies. All results of database search were merged in a single file for monitoring devices after the duplicates from the citation list were removed using the Endnote reference management tool. The initial search was performed in October of 2008, and updated in April 2009.

Inclusion criteria were (1): an article measuring medication adherence in clinical trials using both MEMS and SRQs; (2): the correlation coefficients (Pearson correlation coefficient (r_p_) or Spearman correlation coefficient (r_s_)) between the adherence rates measured by 2 different methods were available or could be calculated based on data published in the study reports.

**Figure 1:**
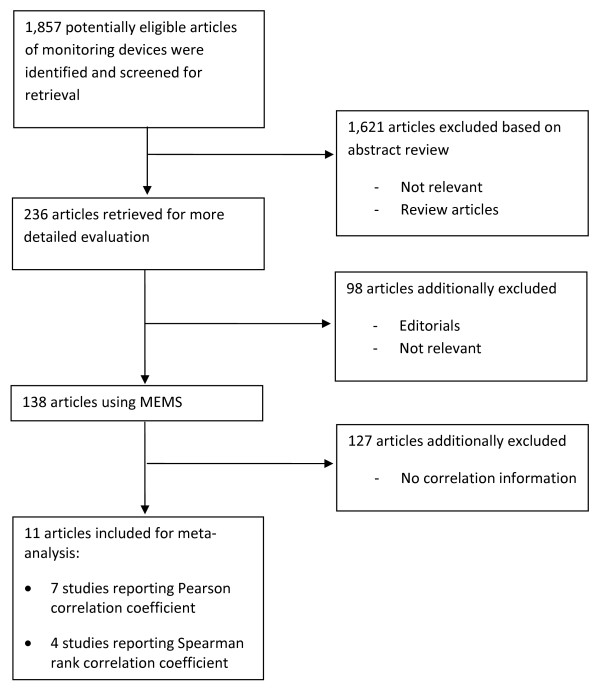
**Flow Chart of Articles Identified and Evaluated during the Study Selection Process**.

Figure 1 presents the flow chart documenting how the research team used to extract the information for study objectives. From the original citations of 1,857 records, 2 research assistants (YK and JL) independently reviewed both files and qualitatively determined "most relevant" "somewhat relevant", and "irrelevant" in accordance with the Quality of Reporting of Meta-analyses (QUOROM) statement, [11] and were re-verified by the Preferred Reporting Items for Systematic Reviews and Meta-Analyses (PRISMA) statements, the latter of which is the most recent standard process for meta-analysis in 2009. Disputes were settled by consensus after reviewing full-text articles. Where discrepancies between investigators occurred for inclusion or exclusion, the principal investigator (LS) was involved to conduct additional evaluation of the study and resolve the dispute.

### Data Abstraction

Data were independently abstracted in duplicate with the standardized protocol and abstraction form. The study characteristics recorded were as follows: 1) first author's name; 2) year of publication; 3) disease status of participants; 4) sample size; 5) mean age (year); 6) duration of trials (month); 7) SRQ names if available or anonymous if a specific name is unavailable in the article; 8) adherence (%) measured by MEMS; 9) adherence (%) by SRQ; 10) correlation coefficient and relative information, including p-value, 95% confidence interval (CI). If data concerning the outcome were missing from an article, the investigators attempted to contact the primary author in order to obtain this missing data.

### Statistical Analysis

This meta-analysis was conducted according to the QUOROM guidelines [11] and PRISMA statements for the conduct and reporting of meta-analyses. Standard methods were used to calculate the pooled variance [12], which were calculated using CIs, p-values, t-statistics, or individual variances for the 2 types of adherence measurements. When a paper reported p < 0.05, p < 0.01, p < 0.001 or NS, we computed standard error of correlation coefficient with p values of 0.025, 0.005, 0.0005, 0.50, respectively, which likely gained a highly conservative estimate of the correlation coefficient [13]. Both fixed-effects and DerSimonian and Laird's random effects models were used to calculate the pooled correlation coefficient [14]. The 2 models approximate each other in the absence of heterogeneity. Heterogeneity was assessed using the chi-square test statistic. The random effect model was selected in this meta-analysis to synthesize correlation coefficient due to heterogeneity among the reviewed studies. We presented data for random-effects models throughout because of the different demographic characteristics, measurement methods, and study durations that were involved in the original trials. Publication bias was examined using the Begg-adjusted rank correlation test based on Kendall's score and Egger regression asymmetric test [15]. Two subgroup *post-hoc *meta-analyses (studies reporting Pearson correlation coefficient and Spearman rank correlation coefficient; HIV studies vs. non-HIV studies) were also conducted to investigate potential differences, to address these naturally occurring groups in the population of studies. All analyses were conducted in STATA version 10.1 (Stata Corp., College Station, TX). The significance was set at 2-tailed p-values of 0.05.

## Results

### Basic characteristics of studies

Figure 1 presents the flow chart to describe the process of selecting the studies for meta-analysis. Out of 1,857 citations, we selected the SRQ articles using the MEMS as concurrent monitoring methods (n = 138). After restricting the articles with correlation between the 2 methods, we only found 11 articles (7 with r_p _and 4 with r_s_). Table 1 summarizes the basic characteristics of studies investigating the correlation between adherence measured by MEMS and SRQs. Across 11 articles finally included in the meta-analysis [16-26], 7 (63.6%) studies' participants were HIV patients. The sample size of included studies ranged from 26 to 568, 153 on average. The mean age was 42.9 years, with a range of 23 to 62 years. The trial period averaged 4.6 months (range 0.5 to 12 months). The mean of adherence measured by MEMS was 74.9% (range 53.4% to 92.9%), compared to 84.0% by the self-report questionnaires (range 68.35% to 95.0%).

**Table 1:** Basic characteristics of studies investigating the correlation between adherence rates measured by MEMS and SRQs

Author	Year	Disease	Sample Size	Age (years)	Duration (months)	Self-Report Questionnaires	MEMS-Monitored Adherence (%)	Self-Report Adherence (%)	Correlation (r_p _or r_s_)
Arnsten J.	2001	HIV	133	43	6	Anonymous	53.4	78.1	0.46
Hugen P.W.	2002	HIV	26	39.9	0.5	VAS	91.1	86	0.73
Walsh J.C	2002	HIV	78	-	6	MASRI	92.9	93.3	0.63
Hamilton G.A.	2003	Hypertension	107	58	-	MOS, Morisky, VAS	58.38	81.05	0.26
Oyugi J.H.	2004	HIV	36	35	3	AACTG	90.9	93.5	0.87
Fletcher C.V.	2005	HIV	258	40	12	AACTG	64	82	0.24
Halkitis P.	2005	HIV	300	42	-	Anonymous	90	95	0.32
Jasti S.	2006	Iron deficiency	51	23	-	Anonymous	68.1	76.5	0.35
Byerly M.J.	2008	Schizophrenia	61	44.3	6	BARS	66.81	68.35	0.59
Lu M.	2008	HIV	568	42	1	Anonymous	69.8	78.8	0.55
Zeller A.	2008	Hypertension Diabetes Dysdipidemia	66	62	2.5	ASRQ	79	91.3	0.29

BARS: Brief adherence rating scale; AACTG: Adult AIDS clinical trials group adherence instrument; MEMS: Medication event monitoring systems; MOS: Medical outcomes study; Morisky: Morisky adherence rating scale; VAS: Visual analog scale; MASRI: Medication adherence self-report inventory; ASRQ: Adherence self-report questionnaire; Anonymous: A questionnaire without a specific name in a reviewed article.

The correlation between adherence measured by MEMS and self-report questionnaires ranged from 0.24 to 0.87 for the 11 articles. We found 7 (63.6%) articles reporting Pearson correlation coefficient (r_p_) [17,19-22,24,26] and 4 (36.4%) using Spearman rank correlation coefficient (r_s_) [16,18,23,25].

### Meta-analysis Results

**Figure 2:**
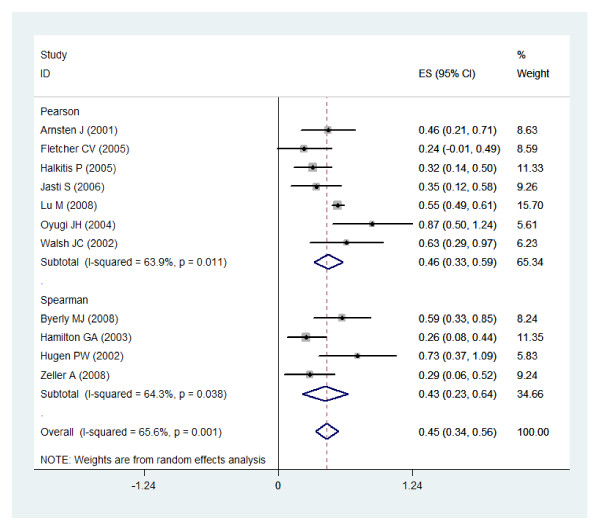
**Correlation coefficients between adherences measures by MEMS and self reported questionnaires and corresponding 95% confidence intervals by study and pooled**.

Figure 2 presents the combined correlation coefficient for 11 studies was 0.45 (p = 0.001, 95% CI: 0.34-0.56). The subgroup meta-analysis on the studies reporting Pearson correlation coefficient and Spearman rank correlation coefficient showed the pooled correlation coefficient 0.46 (p = 0.011, 95% CI: 0.33-0.59) and 0.43 (p = 0.038, 95% CI: 0.23-0.64), respectively. Additionally, another subgroup meta-analysis on HIV patients in the 7 reviewed studies found the pooled correlation coefficient 0.51 (p = 0.014, 95% CI: 0.37-0.64) and non-HIV studies found the pooled correlation coefficient 0.45 (p = 0.001, 95% CI: 0.34-0.56).

The test for heterogeneity among the reviewed studies showed statistically significance in both categories (both p-values < 0.05) and the overall analysis (p = 0.001). Given the heterogeneity statistics presented, we only reported the results of the random-effects models as appropriate models for combining the individual studies.

As to publication bias, the Egger test showed the intercept in the regression of the standardized effect estimates against their precision was -0.75 (p = 0.40, 95% CI: -2.69-1.19) while the Begg test showed a marginally statistical significance (p = 0.052).

## Discussion

This is the first study to our best knowledge to quantify the correlation between the MEMS and SRQs for measuring adherence. We only found a small number of studies which have met the inclusion criteria for meta-analysis. We have found at least moderate correlation using a meta-regression model to pool the correlation coefficients from a total of 11 studies. These findings are consistent with previous studies on the moderate-to-high correlation of self-report with other measures of medication adherence [8-10,27].

The systematic measurement of medication adherence is not routinely performed in outpatient settings due to a lack of reliable, convenient, economical methods for measuring adherence. The key advantages and limitations of various methods have been well summarized in the literature [28]. The selection of medication adherence measures should tailor to the goals and resources available for the intended use and attributes of each type of measures. The 2 methods (MEMS and SRQs) collect different sets of information using different approaches and perspectives. When used together, the 2 methods complement each other giving confidence to the results, and tend to support the same conclusion. The meta-analysis summarizes and advances the field of adherence research through a side-to-side examination on two types of measurements within a study. Our finding of the pooled correlation coefficient of approximate 0.45 supports the need of multiple measures in the future adherence research because neither the MEMs nor SRQs can replace each other.

Furthermore, we have found that most of SRQs used in the meta-analysis were generic measures for medication adherence. For example, among these questionnaires, the Adult AIDS Clinical Trials Group (AACTG) instruments were most frequently used to evaluate clinical interventions, including the efficacy of drugs and drug combinations for treating HIV infection and HIV-associated illnesses [29]. This is a standard self-administered questionnaire based on previous research on adherence. The questionnaire has been in use for over 10 years and patients demonstrated high satisfaction with its length [30,31]. Similarly, the Morisky Scale is widely used to measure medication adherence in various populations (e.g., asthma [32], cancer [33], osteoporosis [34]). It was originally developed to measure hypertension and demonstrated high concurrent and predictive validity with regard to blood pressure control. The 4 items scale and its modified versions: 8- and 5-item scales are relatively simple to use and could be utilized to measure adherence [35,36]. The Medication Adherence Self-Report Inventory (MASRI) is a 12-item questionnaire originally developed for HIV [17] and systemic lupus [37]. However, in contrast to those well-known SRQs, most of the reviewed anonymous questionnaires (4 studies) also found low correlation with MEMS. Therefore, the validity of these anonymous questionnaires was not satisfactory for further development.

These findings must be interpreted in the context of the methodological weaknesses of this study, particularly for the heterogeneity of SRQs in the limited number of included studies. First, some studies have different definitions of adherence, in addition to the variations in study populations, disease states, and study duration. For example, most studies were in HIV patients where adherence is very high. In contrast, for 2 studies that examined non-symptomatic disease such as hypertension, correlation was low. Relatively recent methodological work has been published to assess adherence-response relationships, particularly when adherence is subject to measurement error [38,39]. Secondly, the information on some SRQs is limited in the study reports, even without a specific name for the SRQs in 4 articles. Thirdly, 2 simplistic correlation measures, Pearson correlation coefficients and Spearman correlation coefficients, have been used in the meta-analysis. With the focus on the correlation coefficients, we had an implicit assumption that the association between electronically measured and self-reported adherence rates is linear. Obviously, a non-linear association is possible in the true association for research in the future. Additionally, we have tested the heterogeneity among the studies with a finding of significance. To address the issue of heterogeneity, which is quite common in meta-analysis, we have adopted random-effect models in the meta-analysis due to heterogeneity. We have also done two subgroup analyses to explore some possible influences of heterogeneity. The results of subgroup analyses did not find substantial differences because the results of 95% CI were overlapping for the pooled estimates. Lastly, measuring the level of agreement (not just association) between the MEMS and questionnaire data should be considered in future studies. The Pearson product-moment correlation is a measure of association, not agreement. Perhaps we may also extract an indicator such as the intraclass correlation.

Other limitations should also be mentioned. Although the authors have made attempts to identify all available studies for meta-analysis, there could have been studies that were missed. For example, a recent study was excluded due to the use of different measure of correlation coefficient Kendall tau [27]. Inclusion of other self-reported methods such as diary, claims data, and clinical opinion could potentially be explored in the future. Lastly, the generalizability of the study results is limited as majority of the studies identified as measuring adherence were in HIV and few were in hypertension, schizophrenia and diabetes.

## Conclusion

Based on the pooled estimate using meta-analysis, at least moderate correlation was found between adherences measured by MEMS and SRQs. Therefore, SRQs provide a good estimate of patient medication adherence. If possible, MEMS and SRQs should be used complementarily to get accurate measure for patient adherence.

## Competing interests

Systematic review and meta-analysis were funded by Eli Lilly and Company. This manuscript reflects the opinion of the authors. The authors declare that they have no other competing interests.

## Authors' contributions

LS was the principal investigator (PI) for the project. He conceived of the study, participated in its design, the analytical plan, and the interpretation of the results, and was lead in writing the manuscript. JL performed the statistical analyses, and participated in the design of the study, the analytical plan, and the interpretation of the results. PW assisted the PI on the literature search. VF was the consultant for the project and participated in the interpretation of the results. AK and MP were employees with Eli Lilly and Company, which provided the research contract to the University, and participated in the study conceptualization, study design, analytical plan, interpretation of the results, and manuscript preparation. Part of the study results have been presented in the International Society for Pharmacoeconomics and Outcomes Research (ISPOR) Annual Meeting 2009. Some comments of anonymous reviewers were integrated in the final version. All authors have read and approved the final manuscript.
